# Early EASIX and Short-Term Mortality After Acute Myocardial Infarction: A Dual-Cohort Prognostic Study

**DOI:** 10.3390/jcm15176820

**Published:** 2026-09-03

**Authors:** Yanlong Zhao, Wenyang Liu, Haodong Jiang, Yuanyuan Zhao, Shuai Wang, Qicheng Yu, Jing Zeng, Shan Xie, Jiatong Li, Zhi Liu

**Affiliations:** 1 Department of Emergency, Xuanwu Hospital, Capital Medical University, 45 Changchun Street, Xicheng District, Beijing 100053, China; 2 Department of Proctology, Guang’anmen Hospital, China Academy of Chinese Medical Sciences, Beijing 100053, China; 3 Department of Medical Care, Beijing Luhe Hospital, Capital Medical University, Beijing 100069, China

**Keywords:** acute myocardial infarction, endothelial activation and stress index, mortality, GRACE score, risk stratification, MIMIC-IV

## Abstract

**Background/Objectives**: The Endothelial Activation and Stress Index (EASIX) combines lactate dehydrogenase, creatinine, and platelet count in a three-variable laboratory index. We evaluated its association with early mortality after acute myocardial infarction (AMI) and its incremental prognostic information beyond the Global Registry of Acute Coronary Events (GRACE) score. **Methods**: This retrospective study included a primary hospital cohort of 4167 patients with AMI and an independent MIMIC-IV cohort of 3183 patients with AMI requiring intensive care. EASIX was calculated from the first component measurements within 24 h after admission and log_2_-transformed. Cox models assessed 30-day mortality and changes in the association over time. Incremental performance beyond GRACE and sex was examined in the primary cohort. **Results**: Thirty-day mortality was 3.3% in the primary cohort and 22.9% in MIMIC-IV. Each doubling of EASIX was associated with higher 30-day mortality after adjustment for GRACE and sex in the primary cohort (hazard ratio [HR], 1.25; 95% CI, 1.13–1.38) and after clinical adjustment in MIMIC-IV (average HR, 1.40; 95% CI, 1.36–1.45). The association was strongest near admission and remained consistent across 6, 12, and 24 h exposure windows. Adding EASIX to GRACE modestly improved discrimination, whereas calibration and decision-curve net benefit changed little. **Conclusions**: Early EASIX was independently associated with short-term mortality across two clinically distinct AMI cohorts and provided complementary prognostic information beyond GRACE. Its three laboratory components offer a low-burden summary of early systemic physiological stress.

## 1. Introduction

Acute myocardial infarction remains a major cause of early death despite advances in reperfusion and evidence-based secondary prevention [1,2]. Early risk is concentrated around presentation, when clinicians determine monitoring intensity, invasive management, and level of care. The GRACE score is the best-established tool for this purpose and retains strong discrimination in contemporary acute coronary syndrome populations [3,4]. Its calculation, however, draws on eight variables spanning demographics, vital signs, clinical status, electrocardiography, and biomarkers. A laboratory index available from early testing could complement this broader assessment by providing an automatically calculated signal of systemic physiological stress.

The acute response to myocardial infarction extends beyond cardiomyocyte necrosis. Microvascular injury, inflammation, and haemodynamic compromise accompany tissue injury and contribute to early organ dysfunction [5,6]. EASIX combines lactate dehydrogenase, creatinine, and platelet count and was initially developed to assess complications and mortality after allogeneic stem-cell transplantation [7]. In AMI, these three laboratory variables summarize complementary features of the acute systemic response in a single calculation.

Higher EASIX has since been associated with adverse outcomes in coronary artery disease, critically ill AMI, and non-ST-segment-elevation myocardial infarction [8,9,10]. What remains uncertain is whether an admission value provides reproducible early risk information across clinical settings and adds information to an established comprehensive score. We therefore examined the association of early EASIX with short-term mortality in a broad hospital cohort and an independent AMI intensive-care cohort, characterized how the association changed after admission, and assessed its incremental performance beyond GRACE.

## 2. Materials and Methods

### 2.1. Study Design and Data Sources

We conducted a retrospective dual-cohort study using a hospital-based AMI cohort from Xuanwu Hospital, Capital Medical University, as the primary cohort and MIMIC-IV version 3.0 as an independent external reproducibility cohort. AMI was defined according to the Fourth Universal Definition of Myocardial Infarction [11]. The study was conducted in accordance with the Declaration of Helsinki and approved by the Ethics Committee of Xuanwu Hospital, Capital Medical University (approval No. KS2022161-1). The requirement for informed consent was waived because of the retrospective design and use of routinely collected clinical data.

MIMIC-IV contains deidentified electronic health-record data from Beth Israel Deaconess Medical Center between 2008 and 2022 [12]. Use of these data did not require additional informed consent. Access was granted after completion of the PhysioNet credentialing process, including Collaborative Institutional Training Initiative certification (record number 63998837), and agreement to the data-use terms.

### 2.2. Study Population and Cohort Assembly

Patients were eligible for the primary cohort if they were admitted with AMI between January 2016 and July 2024. For patients with more than one eligible admission, only the first was retained as the index admission. Of 4290 admission records, 27 later or repeated admissions, 7 records without an evaluable admission date, and 89 patients without follow-up were excluded, leaving 4167 patients. STEMI status was obtained from the index-admission record. A missing EASIX component was not an exclusion criterion; missing baseline values were handled according to the prespecified procedure described below.

**Figure 1:**
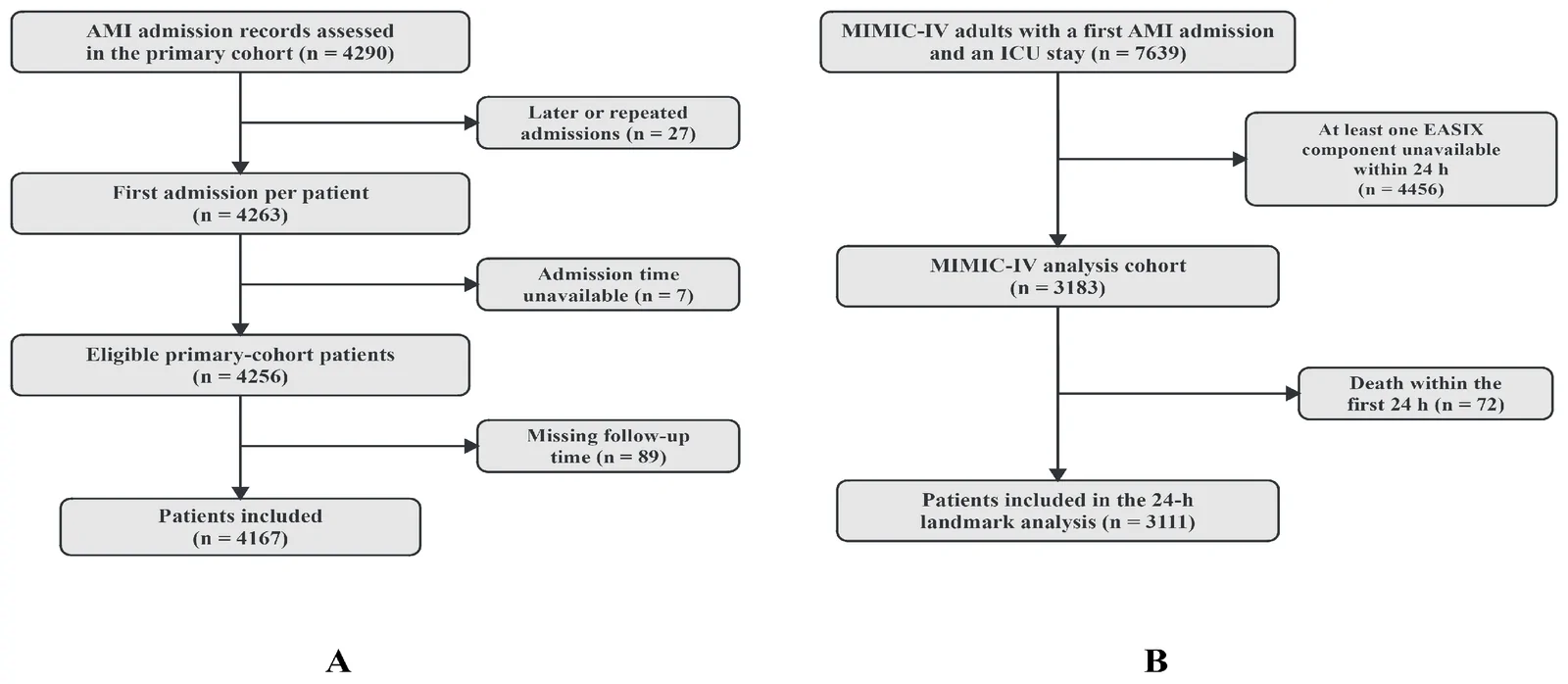
Study flow in the primary and MIMIC-IV cohorts. (**A**) shows construction of the primary cohort. (**B**) shows construction of the independent MIMIC-IV reproducibility cohort and the 24 h landmark analysis. EASIX, Endothelial Activation and Stress Index; ICU, intensive care unit.

For MIMIC-IV, patients were eligible if they were at least 18 years old, had an AMI diagnosis identified from International Classification of Diseases codes, and were admitted to an intensive care unit during the hospitalization. STEMI status was identified from diagnosis-code phenotypes. No independent central adjudication of STEMI/NSTEMI classification was performed in either cohort. The first eligible hospital admission and first intensive care unit stay were retained for each patient. No minimum intensive care length of stay was required. Of 7639 eligible patients, 3183 had valid measurements of all three EASIX components within 24 h after hospital admission and formed the external reproducibility cohort. The other 4456 patients were retained for the selection analysis. Deaths within the first 24 h remained in the primary analysis and were excluded only from the corresponding landmark analysis (Figure 1; Supplementary Table S6).

### 2.3. EASIX

For both cohorts, baseline was hospital admission. Lactate dehydrogenase, creatinine, and platelet count were defined as the first valid measurements obtained within 24 h after admission. Lactate dehydrogenase was expressed in U/L, creatinine in mg/dL, and platelet count in 10^9^/L. Enzymatic creatinine in the primary cohort was converted from μmol/L to mg/dL by division by 88.4. EASIX was calculated using the original formula [7]. EASIX = lactate dehydrogenase (U/L) × creatinine (mg/dL)/platelet count (10^9^/L). Because EASIX was right-skewed, the primary exposure was log_2_(EASIX), so estimates represent the relative change in hazard per 2-fold higher EASIX. Tertiles were used for descriptive and Kaplan–Meier analyses. Cut points derived in the primary cohort (0.874 and 1.643) were applied unchanged to MIMIC-IV and used to describe risk gradients and Kaplan–Meier curves.

### 2.4. Outcomes

The primary outcome was all-cause mortality within 30 days after hospital admission. In the primary cohort, mortality was ascertained from the electronic health record and structured follow-up, including deaths occurring after discharge. In MIMIC-IV, hospital death records and linked death dates were used. All-cause mortality within 365 days and mortality from day 31 through day 365 among 30-day survivors were additionally evaluated in both cohorts. Follow-up was administratively censored at the relevant horizon. Landmark analyses excluded deaths that occurred before the end of each EASIX measurement window and measured subsequent mortality through day 30.

### 2.5. Covariates and GRACE Score

Covariates were selected before modeling according to clinical relevance and availability. The primary-cohort clinical model included age, sex, ST-segment-elevation myocardial infarction, diabetes, hypertension, prior myocardial infarction, prior stroke, atrial fibrillation, and Killip class. The MIMIC-IV clinical model included age, sex, ST-segment-elevation myocardial infarction, heart failure, diabetes, atrial fibrillation, and hypertension. The GRACE score was recalculated from admission variables after missing-data processing using the published model; sex was included separately in GRACE-adjusted analyses [4].

### 2.6. Missing Data

Among 138 candidate baseline variables in the primary cohort, 29 with more than 20% missingness or no observations were excluded. Missing values in retained variables were imputed using missForest [13]. Identifiers, dates, outcomes, and follow-up times were not imputed. Missingness for key analysis variables and patient-level imputation are reported in Supplementary Table S2. The sensitivity analysis was restricted to patients with measured lactate dehydrogenase, creatinine, and platelet count. EASIX components were not imputed in MIMIC-IV.

### 2.7. Statistical Analysis

Continuous variables were summarized as medians with interquartile ranges and categorical variables as counts with percentages. EASIX tertiles were compared using the Kruskal–Wallis, chi-square, or Fisher’s exact test, as appropriate. Kaplan–Meier curves were compared with the log-rank test.

Cox regression estimated associations between log_2_(EASIX) and mortality. Models were unadjusted, adjusted for age and sex, and adjusted for the prespecified clinical covariates; the primary cohort also included a GRACE- and sex-adjusted model. Proportional-hazards assumptions were assessed with Schoenfeld residuals, and time-varying HRs were estimated when required. Restricted cubic splines with four prespecified knots assessed dose–response patterns. A joint model containing log_2_-transformed lactate dehydrogenase, creatinine, and platelet count was compared with the fixed EASIX model using likelihood-ratio tests and Akaike information criteria.

Incremental performance for 30-day mortality was evaluated in the primary cohort by comparing logistic models containing GRACE and sex with and without log_2_(EASIX). Discrimination was assessed using the area under the receiver operating characteristic curve and DeLong’s test [14]; prediction error and calibration were assessed using the Brier score, calibration intercept, calibration slope, and calibration plots. Optimism was estimated with 1000 bootstrap samples. Decision curves used stratified 10-fold out-of-fold predictions across thresholds of 1% to 15%, with 2000 paired bootstrap samples for net-benefit differences [15,16]. Continuous NRI and IDI were exploratory; category-based NRI was not calculated because no clinically actionable risk categories were prespecified [17,18].

Sensitivity analyses examined 6, 12, and 24 h exposure windows and corresponding landmarks, 365-day and day 31–365 mortality, additional adjustment for coded chronic kidney disease, and restriction to measured EASIX components. Included and excluded MIMIC-IV patients were compared using absolute standardized mean differences. Component availability, sampling times, and measurement synchrony were also summarized. Spearman correlation and variance inflation factors were used to assess the relation between EASIX and GRACE.

Prespecified subgroup analyses considered age, sex, ST-segment-elevation status, diabetes, and Killip class. Heterogeneity was assessed using interaction terms with Benjamini–Hochberg adjustment. Tests were two-sided, with *p* < 0.05 considered statistically significant; subgroup findings were based on adjusted interaction *p* values. Analyses were performed using R software (version 4.5.2; R Foundation for Statistical Computing, Vienna, Austria) and followed the STROBE statement [19].

## 3. Results

### 3.1. Study Populations

The primary cohort included 4167 patients; 138 (3.3%) died within 30 days and 196 (4.7%) within 365 days. Of the 138 deaths within 30 days, 132 occurred during the index hospitalization and six were captured through structured follow-up after discharge. Median age was 63 years (interquartile range, 55–71), 3215 patients (77.2%) were men, and 2685 (64.4%) had STEMI. Median EASIX was 1.17 (interquartile range, 0.76–2.10). Higher EASIX tertiles were associated with lower systolic blood pressure and left ventricular ejection fraction, more frequent STEMI and higher Killip class, higher lactate dehydrogenase and creatinine, lower platelet counts, and higher GRACE scores (Table 1).

Values are median [interquartile range] or *n* (%). *p* values were calculated using the Kruskal–Wallis test for continuous variables and the chi-square or Fisher’s exact test for categorical variables, as appropriate. Tertiles were defined using cut points of 0.874 and 1.643. EASIX, Endothelial Activation and Stress Index; GRACE, Global Registry of Acute Coronary Events; LDH, lactate dehydrogenase; LVEF, left ventricular ejection fraction.

The MIMIC-IV analysis cohort included 3183 patients; 728 (22.9%) died within 30 days. Median EASIX was 2.38 (interquartile range, 1.14–5.84), and 1960 patients (61.6%) were in the highest study-derived tertile (Supplementary Table S1). Compared with the 4456 excluded eligible patients, included patients were slightly younger (absolute SMD, 0.132), more often had STEMI (0.117), and had higher 30-day mortality (0.141); absolute SMDs for the other reported characteristics were 0.083 or lower. Complete EASIX availability was limited mainly by LDH testing (41.9%), whereas creatinine and platelet availability exceeded 97%. Median sampling times were 3.50, 3.58, and 3.72 h, respectively; all three measurements were obtained within a 1 h interval in 71.9% of cases and within 6 h in 80.3% of complete cases (Supplementary Table S6).

### 3.2. EASIX and Mortality

**Figure 2:**
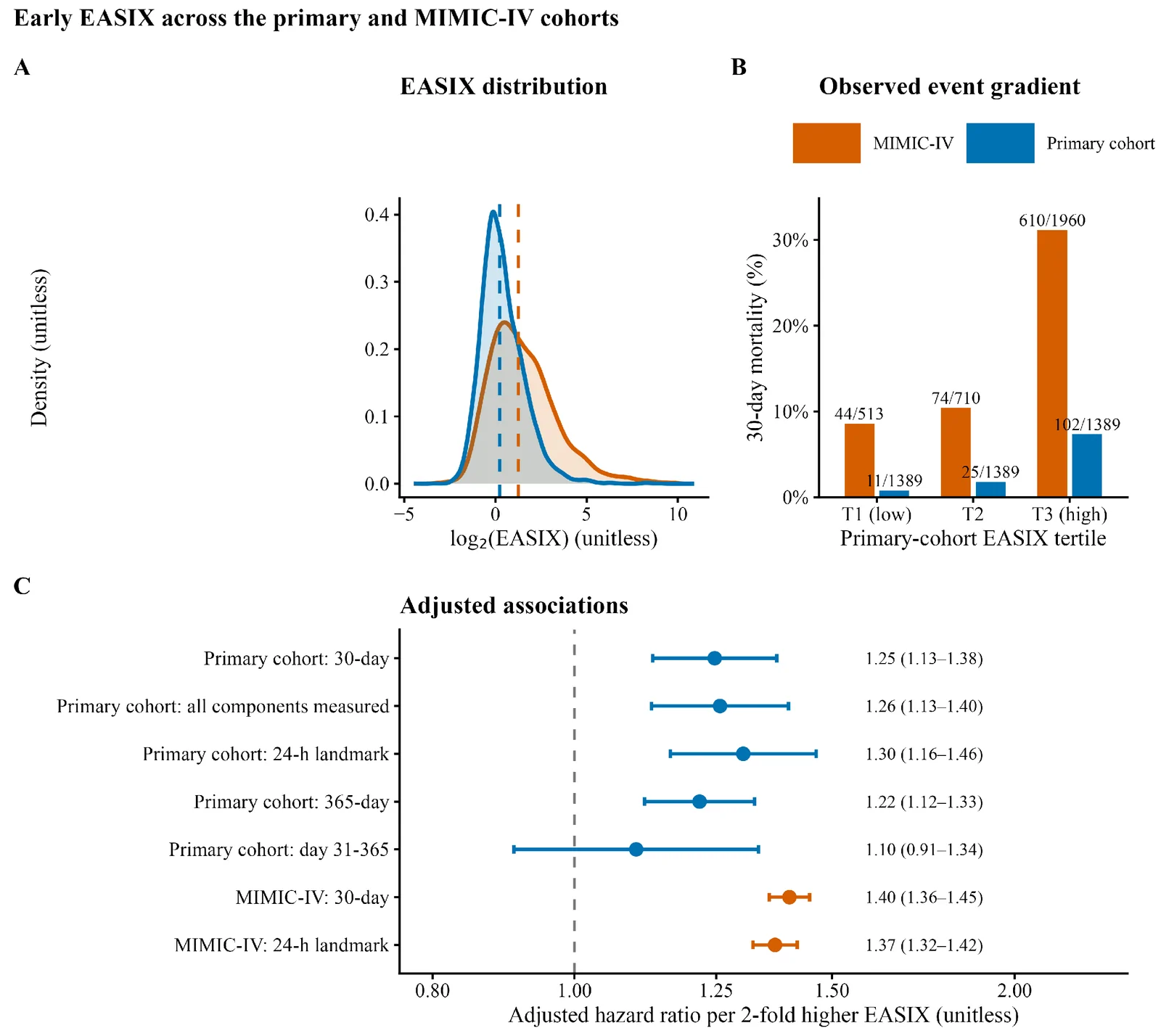
Distribution and mortality associations of early EASIX in the primary and MIMIC-IV cohorts. (**A**) shows the distribution of log_2_-transformed EASIX; dashed vertical lines indicate cohort-specific medians. (**B**) shows observed 30-day mortality across study-derived tertiles; labels show events/total. (**C**) summarizes adjusted hazard ratios per 2-fold higher EASIX. Error bars indicate 95% CIs. Density and hazard ratios are unitless; mortality is shown as a percentage. Primary-cohort estimates were adjusted for GRACE and sex; MIMIC-IV estimates were clinically adjusted.

In the primary cohort, unadjusted 30-day mortality increased from 0.79% (11/1389) in the lowest EASIX tertile to 1.80% (25/1389) in the middle tertile and 7.34% (102/1389) in the highest tertile. The corresponding rates in MIMIC-IV were 8.58% (44/513), 10.42% (74/710), and 31.12% (610/1960) (Figure 2).

Each doubling of EASIX was associated with a 77% higher unadjusted hazard of 30-day death in the primary cohort (hazard ratio [HR], 1.77; 95% CI, 1.65–1.90). The association persisted after clinical adjustment (HR, 1.31; 95% CI, 1.20–1.44) and after adjustment for GRACE and sex (HR, 1.25; 95% CI, 1.13–1.38) (Table 2). EASIX was also associated with 365-day mortality in the clinical model (HR, 1.29; 95% CI, 1.19–1.40) and the GRACE- and sex-adjusted model (HR, 1.22; 95% CI, 1.12–1.33).

Primary-cohort clinical models included age, sex, ST-segment-elevation myocardial infarction, diabetes, hypertension, prior myocardial infarction, prior stroke, atrial fibrillation, and Killip class. GRACE-adjusted models included the recalculated GRACE score and sex. The MIMIC-IV clinical model included age, sex, ST-segment-elevation myocardial infarction, heart failure, diabetes, atrial fibrillation, and hypertension. CI, confidence interval; GRACE, Global Registry of Acute Coronary Events; HR, hazard ratio. Because the EASIX proportional-hazards assumption was not satisfied in MIMIC-IV, its Cox estimates are interpreted as average HRs over 30 days.

The 30-day association was reproduced in MIMIC-IV (clinically adjusted HR, 1.40; 95% CI, 1.36–1.45) (Figure 2). Restricted cubic splines showed overall associations in both cohorts (all *p* < 0.001), with evidence of nonlinearity for 30-day mortality in the primary cohort (*p* = 0.040) and MIMIC-IV (*p* < 0.001), but not for 365-day mortality (*p* = 0.480) (Supplementary Figure S1).

The proportional-hazards assumption was satisfied in the primary cohort but not in MIMIC-IV (Supplementary Table S5). In MIMIC-IV, the adjusted HR per doubling declined from 1.64 (95% CI, 1.53–1.75) at 12 h to 1.24 (95% CI, 1.16–1.31) at day 30 (Supplementary Figure S3). Clinically adjusted average HRs were similar across the 6, 12, and 24 h exposure windows (1.41, 1.41, and 1.40, respectively) and their corresponding landmark analyses (1.40, 1.39, and 1.37) (Supplementary Table S7; Supplementary Figure S4). In MIMIC-IV, 1162 patients died within 365 days; the average adjusted HR was 1.35 (95% CI, 1.32–1.39), decreasing from 1.51 at day 1 to 1.18 at day 365. Among 2455 MIMIC-IV patients alive at day 30, the adjusted HR for mortality from day 31 through day 365 was 1.25 (95% CI, 1.19–1.31). By contrast, among 4029 primary-cohort 30-day survivors, the corresponding HR was 1.10 (95% CI, 0.91–1.34; *p* = 0.322).

### 3.3. Incremental Performance Beyond GRACE

**Figure 3:**
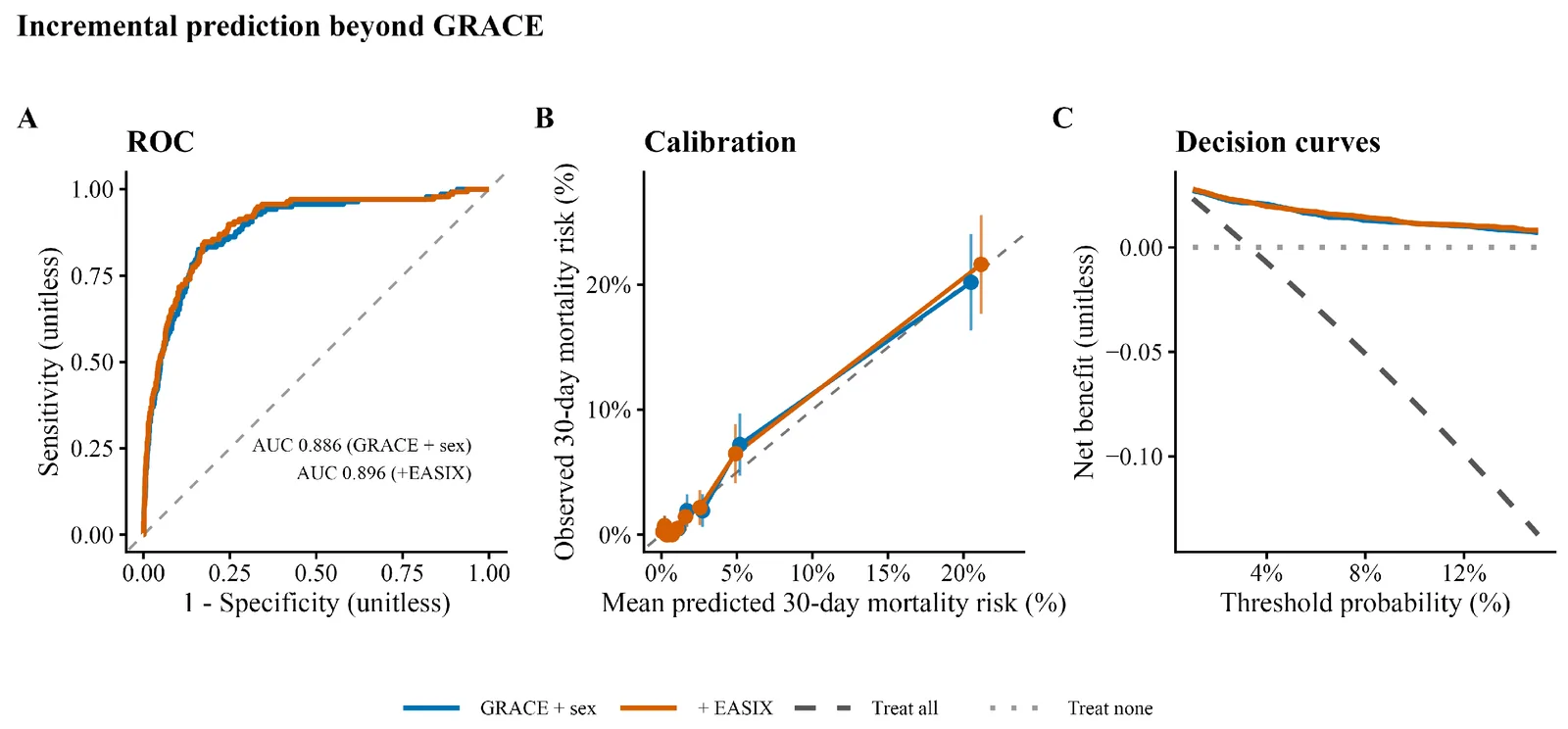
Incremental prognostic performance of EASIX beyond GRACE and sex in the primary cohort. (**A**) shows receiver operating characteristic curves from stratified 10-fold out-of-fold predictions. (**B**) compares observed and predicted 30-day mortality across deciles of predicted risk; error bars indicate approximate 95% CIs. (**C**) shows decision curves across threshold probabilities of 1% to 15%. Sensitivity, 1—specificity, and net benefit are unitless; risks and threshold probabilities are shown as percentages. AUC, area under the receiver operating characteristic curve; GRACE, Global Registry of Acute Coronary Events.

The model containing GRACE and sex had an apparent area under the curve of 0.888 (95% CI, 0.859–0.917), compared with 0.899 (95% CI, 0.872–0.927) after adding log_2_(EASIX). The paired difference was 0.011 (95% CI, 0.002–0.021; *p* = 0.020); optimism-corrected values were unchanged (Table 3). Apparent Brier scores were 0.0271 and 0.0265, respectively, and calibration intercepts were near zero, with slopes near 1 for both models. At 3% and 5% thresholds, cross-validated net-benefit differences were 0.0008 (95% CI, 0.0002–0.0016) and 0.0000 (95% CI, −0.0016 to 0.0015), respectively. Exploratory continuous NRI was 0.239 (95% CI, 0.076–0.412), driven mainly by the non-event component (0.181; 95% CI, 0.153–0.212), and IDI was 0.0188 (95% CI, 0.0053–0.0336) (Figure 3; Supplementary Table S8).

Both models included 4167 patients and 138 deaths within 30 days. Apparent and optimism-corrected estimates were based on 1000 bootstrap samples. Paired areas under the curve were compared using DeLong’s method. Decision-curve and exploratory reclassification results are reported in Supplementary Table S8. AUC, area under the receiver operating characteristic curve; CI, confidence interval.

### 3.4. Sensitivity Analyses and Model Diagnostics

Among 2340 patients with at least one imputed baseline value, 521 had an imputed EASIX component and 619 had an imputed GRACE component (Supplementary Table S2). Restricting the analysis to 3646 patients with all EASIX components measured produced a similarly GRACE- and sex-adjusted association with 30-day mortality (HR, 1.26; 95% CI, 1.13–1.40) (Supplementary Table S3). EASIX and GRACE were moderately correlated (Spearman rho = 0.363), and all variance inflation factors were below 1.30. In MIMIC-IV, additional adjustment for CKD yielded an average HR of 1.41 (95% CI, 1.37–1.46), although the magnitude differed according to coded CKD status (*p* for interaction < 0.001). The unrestricted component model fit better than the fixed EASIX formula in both cohorts. No primary-cohort subgroup interaction remained significant after multiplicity adjustment (all adjusted *p* ≥ 0.170) (Supplementary Tables S4 and S7; Supplementary Figure S2).

## 4. Discussion

Across two clinically distinct AMI cohorts, higher admission EASIX was consistently associated with short-term mortality. In the primary cohort, this association persisted after adjustment for GRACE and sex. Its magnitude was greatest near admission and remained similar across alternative exposure windows, landmark analyses, and the measured-component sensitivity analysis. Adding EASIX to GRACE yielded a small gain in discrimination, with little change in calibration, overall prediction error, or decision-curve net benefit.

Previous studies linked higher EASIX to mortality in critically ill AMI and coronary artery disease [9,10]. Subsequent reports associated EASIX with 1-year mortality beyond GRACE in non-ST-segment-elevation myocardial infarction and with no-reflow or in-hospital mortality after primary percutaneous coronary intervention [8,20,21]. Similar prognostic associations have been reported in chronic heart failure [22]. Our study extends this evidence in three ways. It evaluates a larger hospital-based AMI cohort, reproduces the association in a clinically distinct intensive-care cohort, and shows that the prognostic information is concentrated early after admission. We also assessed incremental performance across discrimination, calibration, reclassification, and net benefit.

EASIX may capture the systemic consequences of acute AMI rather than a single endothelial pathway. Endothelial and microvascular injury can impair reperfusion and promote inflammation and organ hypoperfusion [23,24]. Lactate dehydrogenase reflects cellular injury and tissue hypoperfusion [25,26], whereas creatinine reflects renal reserve and acute haemodynamic stress [27,28]. Platelet count is shaped by thrombotic activity, inflammation, consumption, and marrow response [29,30]. Combining these measures provides a compact summary of tissue injury, renal stress, and platelet response during the acute event. The association changed little after additional adjustment for coded CKD, suggesting that recorded chronic kidney disease did not fully explain it. However, diagnosis codes could not distinguish pre-existing renal dysfunction from evolving acute kidney injury.

The time-varying results clarify what an admission EASIX value represents. In MIMIC-IV, the association was strongest on the first day and then progressively attenuated. Persistent microvascular obstruction and prolonged inflammation are associated with larger infarcts and worse outcomes after reperfused STEMI [31,32], providing a plausible context for this early signal. The absence of an association with day 31–365 mortality among primary-cohort 30-day survivors further supports an acute-state interpretation. The residual long-term association in MIMIC-IV may reflect greater baseline illness and organ dysfunction. Over longer follow-up, ventricular remodeling, recurrent ischaemia, treatment, and secondary prevention assume larger roles [33].

The contrast in absolute mortality between cohorts reflects their different clinical settings. The primary cohort represented a broad hospital AMI population, whereas MIMIC-IV included patients requiring intensive care and early laboratory testing [12]. Within MIMIC-IV, complete early EASIX was determined mainly by LDH testing, and included patients who had higher observed mortality than those without all three components. The MIMIC-IV results therefore provide reproducibility evidence in a more intensively tested AMI-ICU population across a marked shift in baseline risk.

The unrestricted component models fit both cohorts better than the fixed EASIX formula. This pattern is compatible with partly nonlinear relationships between renal and platelet measures and cardiovascular outcomes [34,35,36]. It also reflects a trade-off between statistical fit and implementation. Dataset-specific component weights can improve fit, whereas the fixed formula preserves standardization and transparency. EASIX therefore sacrifices some within-dataset flexibility to provide a portable calculation that requires no coefficient re-estimation. Its associations across heterogeneous cardiovascular populations support this property [37].

EASIX and GRACE captured overlapping but non-identical dimensions of early risk. GRACE remains the benchmark because it integrates age, haemodynamics, heart-failure severity, renal function, electrocardiography, biomarkers, and presentation characteristics [38,39]. EASIX uses only three laboratory measurements and can be calculated automatically once these results are available. Their moderate correlation and low variance inflation factors support complementarity rather than redundancy. Adding EASIX to a baseline AUC of 0.888 increased discrimination by 0.011 points. However, Brier score and net benefit changed little, and the exploratory NRI was driven mainly by non-events [15,40]. These results indicate that EASIX adds measurable prognostic information, although the absolute gain in decision support is small. This profile supports evaluation of EASIX as an automated adjunctive risk flag alongside GRACE. Serial EASIX measurements have retained prognostic information in transplantation cohorts [41,42].

### 4.1. Clinical Implications

When its three laboratory components are available, EASIX can be calculated automatically without further data collection. Its reproducible association across cohorts and measurable incremental information support its evaluation as an adjunctive early risk signal in AMI. Prospective studies should assess automated reporting, serial change, and prespecified action thresholds.

### 4.2. Study Limitations

The findings should be interpreted in light of the retrospective, single-center design of the primary cohort. Residual confounding remains possible. MIMIC-IV represents a selected intensive-care population with complete early LDH, creatinine, and platelet measurements. Reperfusion strategy, procedural success, medication use, peak troponin, and direct endothelial biomarkers could not be harmonized across cohorts. CKD was identified from diagnosis codes, and the analysis used only the first admission EASIX value. Multicenter prospective studies with serial sampling are needed to assess transportability and clinically actionable thresholds.

## 5. Conclusions

Admission EASIX, calculated from three laboratory measurements, was independently associated with early mortality across hospital and intensive-care AMI settings. Its association was strongest near presentation, and it provided a small but measurable increase in discrimination beyond GRACE. These findings support EASIX as a low-burden adjunctive marker of acute systemic physiological stress in AMI.

## Figures and Tables

**Table 1:** Baseline characteristics of the primary cohort according to EASIX tertiles.

Characteristic	Overall (*n* = 4167)	T1 (Low) (*n* = 1389)	T2 (*n* = 1389)	T3 (High) (*n* = 1389)	*p* Value
**Demographics and haemodynamics**					
Age, years	63 [55–71]	61 [54–68]	64 [57–72]	64 [55–74]	<0.001
Male sex	3215 (77.2)	977 (70.3)	1101 (79.3)	1137 (81.9)	<0.001
Heart rate, beats/min	76 [68–86]	75 [68–83]	75 [66–84]	78 [70–88]	<0.001
Systolic blood pressure, mmHg	129 [116–144]	134 [122–146]	130 [117–145]	124 [111–139]	<0.001
**Myocardial infarction presentation and severity**					
ST-segment-elevation myocardial infarction	2685 (64.4)	660 (47.5)	903 (65.0)	1122 (80.8)	<0.001
Killip class 1	2646 (63.5)	1019 (73.4)	932 (67.1)	695 (50.0)	<0.001
Killip class 2	1103 (26.5)	275 (19.8)	346 (24.9)	482 (34.7)	
Killip class 3	313 (7.5)	84 (6.0)	93 (6.7)	136 (9.8)	
Killip class 4	105 (2.5)	11 (0.8)	18 (1.3)	76 (5.5)	
Cardiopulmonary resuscitation	52 (1.2)	4 (0.3)	11 (0.8)	37 (2.7)	<0.001
Cardiogenic shock	4 (0.1)	0 (0.0)	3 (0.2)	1 (0.1)	0.326
**Medical history**					
Hypertension	2504 (60.1)	849 (61.1)	836 (60.2)	819 (59.0)	0.507
Diabetes	1514 (36.3)	527 (37.9)	492 (35.4)	495 (35.6)	0.310
Prior myocardial infarction	120 (2.9)	26 (1.9)	34 (2.4)	60 (4.3)	<0.001
Prior stroke	260 (6.2)	58 (4.2)	72 (5.2)	130 (9.4)	<0.001
Atrial fibrillation	154 (3.7)	19 (1.4)	45 (3.2)	90 (6.5)	<0.001
**Laboratory and cardiac assessment**					
Lactate dehydrogenase, U/L	290 [204–505]	197 [165–228]	286 [229–370]	639 [436–947]	<0.001
Creatinine, mg/dL	0.82 [0.70–0.94]	0.75 [0.64–0.84]	0.83 [0.72–0.93]	0.90 [0.77–1.16]	<0.001
Platelet count, 10^9^/L	215 [185–257]	236 [209–279]	207 [183–239]	201 [163–245]	<0.001
Left ventricular ejection fraction, %	58.0 [51.0–63.0]	61.4 [56.0–65.0]	59.0 [54.0–63.0]	53.0 [45.0–58.6]	<0.001
**Risk indices**					
EASIX	1.17 [0.76–2.10]	0.64 [0.50–0.76]	1.17 [1.00–1.36]	2.75 [2.10–4.27]	<0.001
GRACE score	142 [120–167]	128 [109–150]	142 [123–167]	157 [134–185]	<0.001

**Table 2:** Association between EASIX and mortality in the primary and MIMIC-IV cohorts.

Model	N	Deaths	HR Per Doubling (95% CI)	*p* Value
**Primary cohort—30-day mortality**				
Unadjusted	4167	138	1.77 (1.65–1.90)	<0.001
Age- and sex-adjusted	4167	138	1.77 (1.64–1.91)	<0.001
Clinical model	4167	138	1.31 (1.20–1.44)	<0.001
GRACE- and sex-adjusted	4167	138	1.25 (1.13–1.38)	<0.001
**Primary cohort—365-day mortality**				
Unadjusted	4167	196	1.70 (1.59–1.82)	<0.001
Age- and sex-adjusted	4167	196	1.69 (1.58–1.82)	<0.001
Clinical model	4167	196	1.29 (1.19–1.40)	<0.001
GRACE- and sex-adjusted	4167	196	1.22 (1.12–1.33)	<0.001
**MIMIC-IV—30-day mortality**				
Unadjusted	3183	728	1.36 (1.32–1.40)	<0.001
Age- and sex-adjusted	3183	728	1.40 (1.36–1.44)	<0.001
Clinical model	3183	728	1.40 (1.36–1.45)	<0.001

**Table 3:** Incremental prognostic performance after adding EASIX to GRACE and sex variables.

Measure	GRACE and Sex	GRACE, Sex, and log_2_(EASIX)	Incremental Result	*p* Value
**Discrimination**				
Apparent AUC (95% CI)	0.888 (0.859–0.917)	0.899 (0.872–0.927)	ΔAUC 0.011 (0.002–0.021)	0.020
Optimism-corrected AUC	0.888	0.899	ΔAUC 0.011	—
**Overall prediction error**				
Apparent Brier score	0.0271	0.0265	ΔBrier −0.0006	—
Optimism-corrected Brier score	0.0272	0.0267	ΔBrier −0.0005	—
**Calibration**				
Corrected calibration intercept	−0.003	0.002	—	—
Corrected calibration slope	0.998	0.995	—	—
**Model fit**				
Likelihood-ratio test for adding log_2_(EASIX)	—	—	χ^2^ = 25.36 (1 df)	<0.001

## Data Availability

MIMIC-IV version 3.0 is available to credentialed users through PhysioNet (https://doi.org/10.13026/hxp0-hg59) under its data-use agreement. Individual-level primary-cohort data are not publicly available because of institutional and ethical restrictions. Deidentified data may be available from the corresponding author on reasonable request, subject to institutional approval and an appropriate data-use agreement.

## Supplementary Materials

The following supporting information can be downloaded at https://www.mdpi.com/article/10.3390/jcm15176820/s1, Table S1: Baseline characteristics of the MIMIC-IV cohort according to primary-cohort EASIX tertiles; Table S2: Missingness and imputation in the primary cohort; Table S3: Sensitivity analyses; Table S4: Comparison of EASIX with component-based models; Table S5: Proportional-hazards assumption tests; Table S6: Selection, availability, and measurement timing in MIMIC-IV; Table S7: Sensitivity analyses for timing, long-term outcomes, and chronic kidney disease in MIMIC-IV; Table S8: Incremental performance, reclassification, and relation to GRACE; Figure S1: Dose-response association between EASIX and mortality; Figure S2: Subgroup analyses in the primary cohort; Figure S3: Time-varying association of EASIX with mortality; Figure S4: MIMIC-IV sensitivity analyses and decision-curve net-benefit differences.
