# Supplementary material for: Early EASIX and Short-Term Mortality After Acute Myocardial Infarction: A Dual-Cohort Prognostic Study
Source: J Clin Med. 2026 Sep 3;15(17):6820. doi: 10.3390/jcm15176820 (PMC13566773; doi:10.3390/jcm15176820)
Supplement: Supplementary file 1 [file jcm-15-06820-s001.zip › jcm-4506618-supplementary.pdf]

Supplementary Material

Early EASIX and Short-Term Mortality After Acute Myocardial Infarction: A Dual-Cohort Prognostic Study

Supplementary Tables S1–S8 and Supplementary Figures S1–S4

Supplementary Tables

Supplementary Table S1. Baseline characteristics of the MIMIC-IV cohort according to primary-cohort EASIX tertiles

Characteristic	Overall (n=3,183)	T1 (low) (n=513)	T2 (n=710)	T3 (high) (n=1,960)	P value
**Demographics**					
Age, years	71.2 [61.9–79.6]	68.2 [60.1–75.4]	72.0 [63.1–79.9]	71.8 [62.1–80.6]	<0.001
Male sex	2,069 (65.0)	284 (55.4)	465 (65.5)	1,320 (67.3)	<0.001
**AMI presentation**					
STEMI	1,771 (55.6)	315 (61.4)	412 (58.0)	1,044 (53.3)	0.001
**Medical history**					
Heart failure	1,667 (52.4)	175 (34.1)	311 (43.8)	1,181 (60.3)	<0.001
Hypertension	2,417 (75.9)	361 (70.4)	559 (78.7)	1,497 (76.4)	0.003
Diabetes	1,384 (43.5)	194 (37.8)	304 (42.8)	886 (45.2)	0.010
Atrial fibrillation	1,188 (37.3)	143 (27.9)	276 (38.9)	769 (39.2)	<0.001
**Laboratory assessment**					
Lactate dehydrogenase, U/L	314 [225–530]	208 [171–250]	248 [209–315]	432 [290–732]	<0.001
Creatinine, mg/dL	1.20 [0.90–2.00]	0.80 [0.70–0.90]	1.00 [0.80–1.30]	1.70 [1.10–2.70]	<0.001
Platelet count, 10^9/L	204 [152–262]	273 [225–336]	218 [185–264]	177 [127–233]	<0.001
**Risk index**					
EASIX	2.38 [1.14–5.84]	0.64 [0.50–0.75]	1.22 [1.03–1.42]	4.60 [2.67–9.61]	<0.001

Supplementary Table S1. Baseline characteristics of the MIMIC-IV cohort according to primary-cohort EASIX tertiles. Values are median [interquartile range] or n (%). Primary-cohort cut points of 0.874 and 1.643 were applied unchanged. Only patients with all three EASIX components measured within 24 h after hospital admission were included. EASIX, Endothelial Activation and Stress Index; LDH, lactate dehydrogenase; STEMI, ST-segment-elevation myocardial infarction.

Supplementary Table S2. Missingness and imputation in the primary cohort

Missingness in key analysis variables

Variable	Missing, n	Missing, %
Baseline left ventricular ejection fraction	536	12.9
Lactate dehydrogenase	466	11.2
Creatinine (enzymatic assay)	444	10.7
Platelet	397	9.5
Killip class	110	2.6
Age	40	0.96
Heart rate	35	0.84
Systolic blood pressure	25	0.60
Diabetes	2	0.05
Hypertension grade	2	0.05

Imputation summary

Metric	Value
Baseline variables evaluated	138
Variables excluded for >20% missingness or no observations	29
Patients with any baseline imputation	2340
Patients with any EASIX component imputed	521
Patients with any GRACE component imputed	619
Retained baseline values missing after imputation	0

Supplementary Table S2. Missingness and imputation in the primary cohort. Key analysis variables with at least one missing value are reported together with the imputation summary. Among 138 baseline variables, 29 with more than 20% missingness or no observations were excluded. Missing values in retained variables were imputed using missForest. Identifiers, dates, outcomes, and follow-up times were not imputed. EASIX and GRACE were calculated after processing their component variables.

Supplementary Table S3. Sensitivity analyses

Analysis	N	Deaths	HR per doubling (95% CI)	P value
**Primary cohort — 30-day mortality**				
All components measured	3646	114	1.26 (1.13–1.40)	<0.001
**Primary cohort — Day 1-30 mortality**				
24-hour landmark	4127	98	1.30 (1.16–1.46)	<0.001
**MIMIC-IV — Day 1-30 mortality**				
24-hour landmark	3111	656	1.37 (1.32–1.42)	<0.001
**Primary cohort — Day 31-365 mortality**				
Conditional survival	4029	58	1.10 (0.91–1.34)	0.322

Supplementary Table S3. Sensitivity analyses. The complete-component analysis was restricted to patients with measured LDH, creatinine, and platelet count. Landmark analyses excluded deaths during the first 24 h. Conditional survival included patients alive at day 30. CI, confidence interval; HR, hazard ratio.

Supplementary Table S4. Comparison of EASIX with component-based models

Associations of individual EASIX components

Component	Adjusted HR per doubling (95% CI)	P value
**Primary cohort — 30-day mortality**		
LDH	1.64 (1.39–1.93)	<0.001
Creatinine	1.09 (0.84–1.41)	0.520
Platelet	1.26 (0.86–1.84)	0.229
**Primary cohort — 365-day mortality**		
LDH	1.45 (1.25–1.67)	<0.001
Creatinine	1.22 (0.99–1.51)	0.060
Platelet	1.12 (0.81–1.54)	0.510
**MIMIC-IV — 30-day mortality**		
LDH	1.57 (1.49–1.65)	<0.001
Creatinine	1.36 (1.26–1.46)	<0.001
Platelet	0.87 (0.80–0.95)	0.001

Model comparison

Cohort and outcome	N	Deaths	LR χ^2 (df)	P value	AIC, EASIX	AIC, components
Primary cohort — 30-day mortality	4167	138	12.89 (2)	0.002	1921.7	1912.8
Primary cohort — 365-day mortality	4167	196	6.53 (2)	0.038	2824.6	2822.1
MIMIC-IV — 30-day mortality	3183	728	34.82 (2)	<0.001	11137.7	11106.8

Supplementary Table S4. Comparison of EASIX with component-based models. LDH, creatinine, and platelet count were entered simultaneously on the $\log_2$ scale with the corresponding clinical covariates. The likelihood-ratio test compared EASIX with the component-based model. Lower AIC indicates better relative fit. AIC, Akaike information criterion; CI, confidence interval; HR, hazard ratio; LDH, lactate dehydrogenase; LR, likelihood ratio.

Supplementary Table S5. Proportional-hazards assumption tests

Cohort and outcome	Adjusted model	EASIX PH test, χ^2 (P value)	Global test, χ^2 (df)	P value
Primary cohort — 30-day mortality	GRACE and sex	1.14 (0.286)	4.22 (3)	0.238
Primary cohort — 365-day mortality	GRACE and sex	1.59 (0.207)	5.81 (3)	0.121
MIMIC-IV — 30-day mortality	Clinical model	21.46 (<0.001)	54.14 (8)	<0.001

Supplementary Table S5. Proportional-hazards diagnostics for the adjusted models. Tests were based on Schoenfeld residuals. The primary-cohort models satisfied the proportional-hazards assumption. Evidence of time dependence in MIMIC-IV prompted time-varying and piecewise analyses. GRACE, Global Registry of Acute Coronary Events; PH, proportional hazards.

Supplementary Table S6. Selection, availability, and measurement timing in MIMIC-IV

**Included versus excluded eligible MIMIC-IV patients**			
**Characteristic**	**Included (n=3,183)**	**Excluded (n=4,456)**	**Absolute SMD**
Age, years	71.2 [61.9–79.6]	72.8 [63.2–82.1]	0.132
Male sex	2,069 (65.0%)	2,785 (62.5%)	0.052
STEMI	1,771 (55.6%)	2,220 (49.8%)	0.117
Heart failure	1,667 (52.4%)	2,216 (49.7%)	0.053
Diabetes	1,384 (43.5%)	1,943 (43.6%)	0.002
Atrial fibrillation	1,188 (37.3%)	1,581 (35.5%)	0.038
Hypertension	2,417 (75.9%)	3,537 (79.4%)	0.083
30-day mortality	728 (22.9%)	768 (17.2%)	0.141
**Availability and timing of EASIX components**			
**Component**	**Available, n/N (%)**	**Sampling time, median [IQR], h**	
LDH	3,199/7,639 (41.9%)	3.50 [1.55–9.06]	
Creatinine	7,463/7,639 (97.7%)	3.58 [1.62–7.63]	
Platelet count	7,411/7,639 (97.0%)	3.72 [1.65–8.18]	
**Measurement synchrony among complete cases**			
**Measure**		**Value**	
Complete cases, n		3,183	
Earliest-to-latest component interval, median [IQR], h		0.00 [0.00–3.31]	
All components at the same recorded time		71.3%	
All components within 1 h		71.9%	
All components within 3 h		74.5%	
All components within 6 h		80.3%	
All components within 12 h		93.0%	

Supplementary Table S6. Selection, availability, and measurement timing in MIMIC-IV. Included patients had all three EASIX components measured within 24 h after hospital admission; excluded patients lacked at least one component. Continuous values are median [interquartile range] and categorical values are n (%). Absolute standardized mean differences describe selection differences without relying on sample-size-dependent P values. Sampling time was measured from hospital admission. EASIX, Endothelial Activation and Stress Index; IQR, interquartile range; LDH, lactate dehydrogenase; SMD, standardized mean difference; STEMI, ST-segment-elevation myocardial infarction.

Supplementary Table S7. Sensitivity analyses for timing, long-term outcomes, and chronic kidney disease in MIMIC-IV

**Window-defined cohorts**				
**Exposure window**	**N**	**Deaths**	**Adjusted HR per 2-fold higher EASIX (95% CI)**	**P value**
6 h	1,990	418	1.41 (1.35–1.46)	<0.001
12 h	2,608	583	1.41 (1.36–1.46)	<0.001
24 h	3,183	728	1.40 (1.36–1.45)	<0.001
**Landmark analyses**				
**Landmark**	**N**	**Deaths after landmark**	**Adjusted HR per 2-fold higher EASIX (95% CI)**	**P value**
6 h	1,981	409	1.40 (1.34–1.45)	<0.001
12 h	2,579	554	1.39 (1.34–1.44)	<0.001
24 h	3,111	656	1.37 (1.32–1.42)	<0.001
**Long-term and CKD sensitivity analyses**				
**Analysis**	**N**	**Deaths**	**Adjusted HR per 2-fold higher EASIX (95% CI)**	**P value**
365-day mortality	3,183	1,162	1.35 (1.32–1.39)	<0.001
Day 31–365 among 30-day survivors	2,455	434	1.25 (1.19–1.31)	<0.001
30-day mortality, additionally adjusted for CKD	3,183	728	1.41 (1.37–1.46)	<0.001
30-day mortality, no CKD	2,135	436	1.48 (1.42–1.54)	<0.001
30-day mortality, CKD	1,048	292	1.30 (1.22–1.38)	<0.001

Supplementary Table S7. Sensitivity analyses for timing, long-term outcomes, and chronic kidney disease in MIMIC-IV. Models were adjusted for age, sex, STEMI, heart failure, diabetes, atrial fibrillation, and hypertension; the CKD-adjusted model additionally included coded CKD. All 30-day MIMIC-IV estimates and the overall 365-day estimate are reported as average hazard ratios because the EASIX proportional-hazards assumption was not satisfied; the day 31–365 conditional-survival model satisfied this assumption. Landmark analyses excluded deaths before the corresponding window ended. CKD was defined by ICD-9 code 585* or ICD-10 code N18*. The EASIX-by-CKD interaction P value was <0.001. CI, confidence interval; CKD, chronic kidney disease; HR, hazard ratio; STEMI, ST-segment-elevation myocardial infarction.

Supplementary Table S8. Incremental performance, reclassification, and relation to GRACE

**Decision-curve net-benefit differences**				
**Threshold probability**	**GRACE + sex**	**+ EASIX**	**Difference (95% CI)**	**Difference per 100 patients (95% CI)**
1%	0.0272	0.0278	0.0006 (0.0001–0.0014)	0.063 (0.011–0.136)
3%	0.0215	0.0223	0.0008 (0.0002–0.0016)	0.081 (0.023–0.157)
5%	0.0183	0.0182	0.0000 (−0.0016 to 0.0015)	−0.003 (−0.163 to 0.147)
10%	0.0115	0.0115	0.0000 (−0.0019 to 0.0017)	0.000 (−0.187 to 0.173)
15%	0.0071	0.0082	0.0011 (−0.0008 to 0.0029)	0.106 (−0.085 to 0.291)

**Exploratory category-free reclassification**	
**Metric**	**Estimate (95% CI)**
Continuous NRI	0.239 (0.076–0.412)
Event component	0.058 (−0.107 to 0.224)
Non-event component	0.181 (0.153–0.212)
IDI	0.0188 (0.0053–0.0336)

**Relation between EASIX and GRACE in the primary cohort**	
**Measure**	**Estimate**
Spearman correlation: $\log_2$(EASIX) with GRACE	0.363
Maximum variance inflation factor in the GRACE-adjusted model	1.284

Supplementary Table S8. Incremental performance, reclassification, and relation to GRACE. Decision-curve results used paired stratified 10-fold out-of-fold predictions; 95% confidence intervals for differences, NRI, and IDI were derived from 2,000 paired bootstrap samples. Continuous NRI and IDI are exploratory category-free measures. The continuous NRI was driven mainly by the non-event component and should not be interpreted as evidence of clinical decision benefit. CI, confidence interval; GRACE, Global Registry of Acute Coronary Events; IDI, integrated discrimination improvement; NRI, net reclassification improvement.

Supplementary Figures

Supplementary Figure S1. Dose-response association between EASIX and mortality

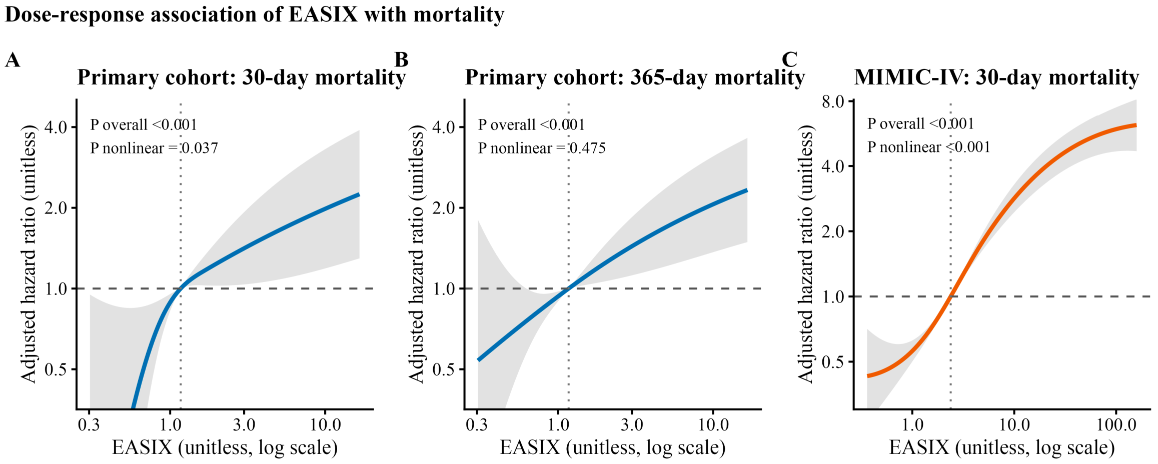

Supplementary Figure S1. Dose-response association between EASIX and mortality. Restricted cubic spline curves show adjusted hazard ratios for 30-day mortality in the primary cohort (A), 365-day mortality in the primary cohort (B), and 30-day mortality in MIMIC-IV (C). Four prespecified knots were used. EASIX and hazard ratios are unitless. Solid lines indicate hazard ratios, shaded areas indicate 95% confidence intervals, horizontal dashed lines indicate a hazard ratio of 1, and vertical dotted lines indicate the reference EASIX value.

Supplementary Figure S2. Subgroup analyses in the primary cohort

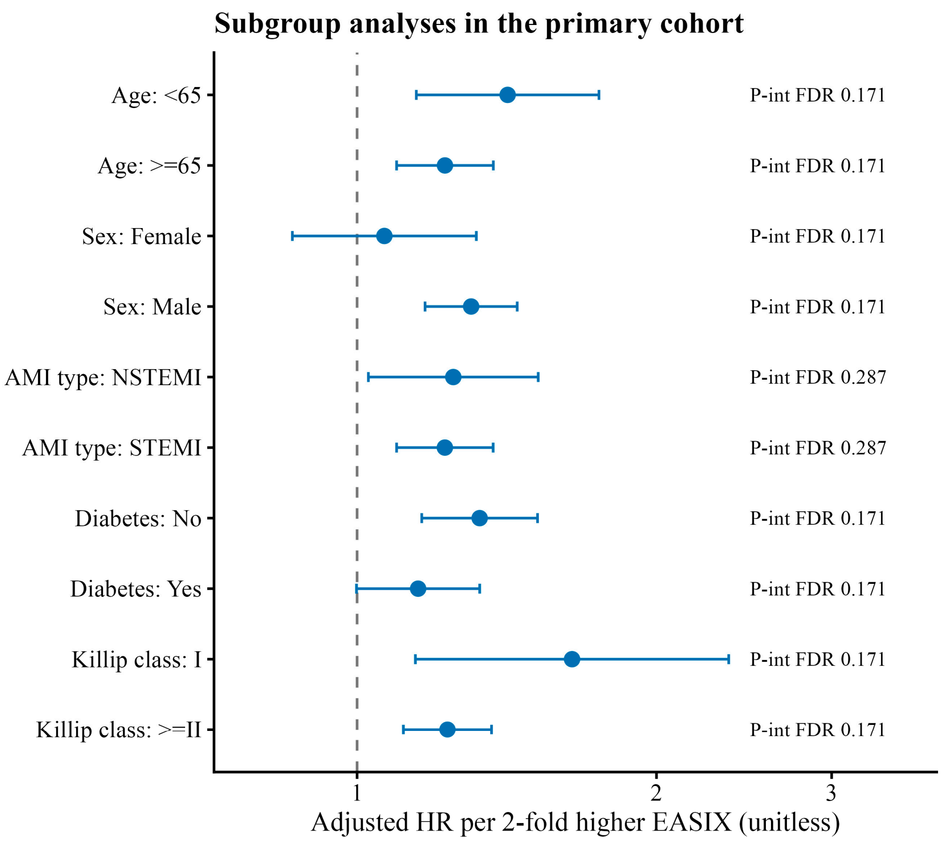

Supplementary Figure S2. Subgroup analyses in the primary cohort. Adjusted hazard ratios per 2-fold higher EASIX are shown by age, sex, STEMI status, diabetes, and Killip class. Hazard ratios are unitless; error bars indicate 95% confidence intervals. Heterogeneity was evaluated with interaction terms and Benjamini–Hochberg adjustment.

Supplementary Figure S3. Time-varying association of EASIX with mortality

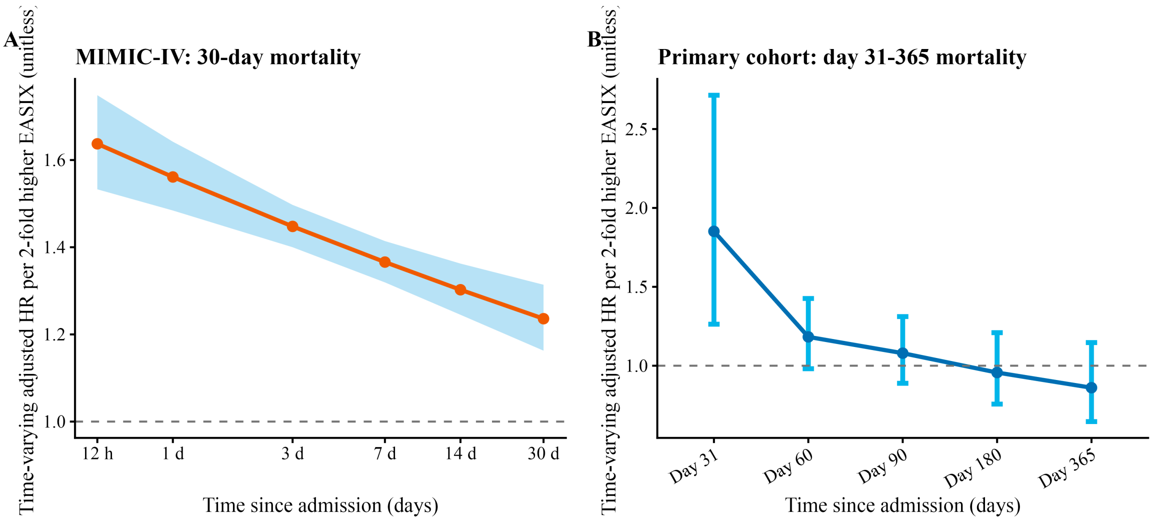

Supplementary Figure S3. Time-varying association of EASIX with mortality. Panel A shows the time-varying clinically adjusted hazard ratio for 30-day mortality in MIMIC-IV. Panel B shows the time-varying GRACE- and sex-adjusted association from day 31 through day 365 among 30-day survivors in the primary cohort. Time is measured in days from admission and hazard ratios are unitless. Lines or points indicate hazard ratios and shaded areas or error bars indicate 95% confidence intervals.

Supplementary Figure S4. MIMIC-IV sensitivity analyses and decision-curve net-benefit differences

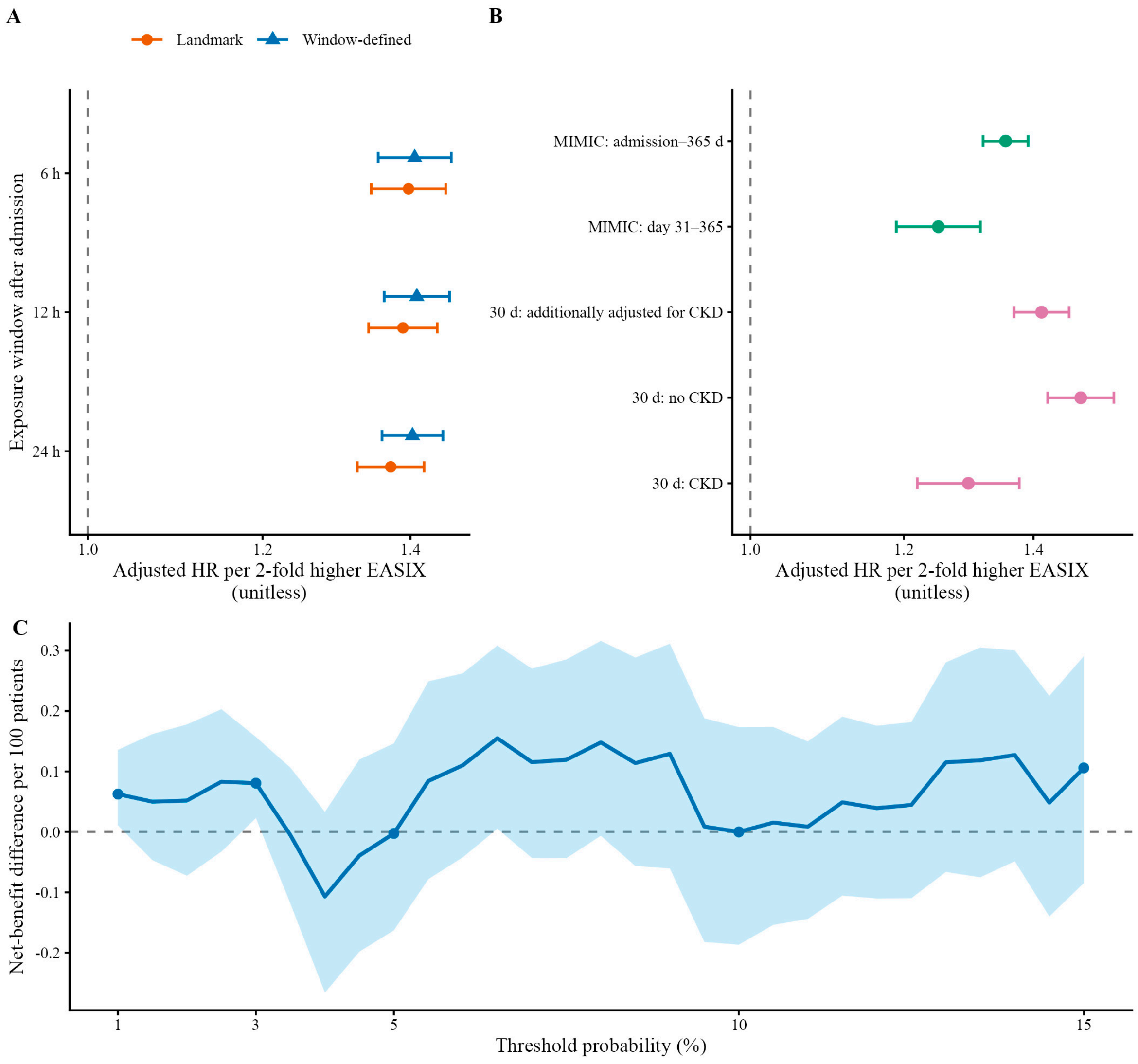

Supplementary Figure S4. MIMIC-IV sensitivity analyses and decision-curve net-benefit differences. Panel A compares clinically adjusted average hazard ratios in MIMIC-IV from 6-, 12-, and 24-h window-defined cohorts and corresponding landmark analyses. Panel B shows clinically adjusted MIMIC-IV long-term and CKD sensitivity estimates. Panel C shows, in the primary cohort, the cross-validated net-benefit difference between GRACE + sex + EASIX and GRACE + sex across threshold probabilities of 1% to 15%; shading indicates the paired-bootstrap 95% confidence interval. Hazard ratios and net benefit are unitless; exposure windows and follow-up are measured in hours or days as labeled, and threshold probabilities are percentages. CI, confidence interval; CKD, chronic kidney disease; EASIX, Endothelial Activation and Stress Index; GRACE, Global Registry of Acute Coronary Events; HR, hazard ratio.
